# Computational study of carbon-doped TiO_2_(B) nanomaterials for improved dye-sensitized solar cells

**DOI:** 10.1038/s41598-026-38897-7

**Published:** 2026-02-10

**Authors:** Herman Heffner, Jorge M. Marchetti, Ricardo Faccio, Ignacio López-Corral

**Affiliations:** 1 Professur für Neuartige Elektronik Technologien, Technische Universität Dresden, Nöthnitzer Str. 61, 01187 Dresden, Germany; 2 Instituto de Química del Sur (INQUISUR, UNS-CONICET), Departamento de Química, Universidad Nacional del Sur, Av. Alem 1253, B8000CPB Bahía Blanca, Argentina; 3 Faculty of Science and Technology, Norwegian University of Life Sciences, Drøbakveien 31, 1430 Ås, Norway; 4 Área Física & Centro NanoMat, DETEMA, Facultad de Química, Universidad de la República, Av. Gral. Flores 2124, 11800 Montevideo, Uruguay

**Keywords:** TiO_2_(B), Carbon doping, Adsorption energy, DFT, N719, DSSC, Chemistry, Materials science, Nanoscience and technology

## Abstract

Surface doping has emerged as a promising approach to enhance the reactivity and optoelectronic properties of titanium dioxide (TiO_2_) and other inorganic oxide semiconductors. This strategy has significant potential to improve the efficiency and long-term stability of dye-sensitized solar cells (DSSCs). The present study employs density functional theory (DFT) calculations to investigate, for the first time, the adsorption behavior of the organometallic N719 dye on pristine and carbon-doped ultrathin TiO_2_(B) films. Initially, the interaction between the N719 dye and the pristine TiO_2_(B) (100) surface is examined, considering various molecular orientations and anchoring configurations. The adsorption energies and the resultant changes in the semiconductor’s electronic structure are determined. Subsequently, the impact of carbon doping on the preferential adsorption configurations is analyzed. The results reveal that the adsorption of the N719 dye is energetically favorable on both the pristine and C-doped TiO_2_(B) (100) surfaces. Notably, all adsorption-related properties are significantly enhanced after carbon doping, with the adsorption energy increasing by up to 300% compared to the undoped surface. This substantial increase in adsorption performance is critical for achieving highly efficient and long-lasting DSSCs.

**Supplementary Information:**

The online version contains supplementary material available at 10.1038/s41598-026-38897-7.

## Introduction

Dye-sensitized solar cells (DSSCs) have emerged as a promising alternative to traditional silicon-based solar cells due to their potential for low-cost production, flexibility, and relatively high efficiency under diffuse light conditions^1–3^. The core of DSSC technology lies in sensitizing a wide-bandgap semiconductor, typically titanium dioxide (TiO_2_), with a photosensitive dye that absorbs sunlight and converts it into electrical energy. This technology has recently achieved 15% efficiency^4^, leaving an extensive margin for future improvements. Among the various dyes used, the ruthenium-based N719 dye has been extensively studied and is known for its high efficiency and stability in DSSC applications^5,6^. The interaction between the dye molecules and semiconductor layers significantly influences the performance of DSSCs. Dye adsorption onto the TiO_2_ surface is such a critical step that cosensitizers were developed to increase the dye-surface interaction^7^. It affects electron injection efficiency, charge recombination rates, and ultimately, the photovoltaic performance of the cell^4^. Modifications of TiO_2_, such as surface defects, have been explored to enhance the adsorption properties of N719 and improve the efficiency of DSSCs^8^. Among all TiO_2_ polymorphs, the bronze phase (B) is garnering increasing attention due to its promising applications in solar cells^9,10^, and lithium-ion batteries^11,12^.

One promising approach to enhance the adsorption and optoelectronic properties of TiO_2_ is through surface doping^13^. This doping can introduce new electronic states within the band gap of TiO_2_, facilitating better charge separation and reducing the recombination of photogenerated electron-hole pairs^14^. Such modification can potentially increase the adsorption energy of dye molecules, leading to improved electron injection and overall efficiency of the DSSCs^15^. For instance, surface treatments such as oxalic acid treatment have been shown to enhance the binding of N719 dye to the TiO_2_ surface, leading to improved electron transfer and device efficiency^16^.

In recent decades, numerous studies have been conducted using organometallic molecules to sensitize various TiO_2_ surfaces, aiming to investigate molecular interactions and their potential applications in DSSCs^17–25^. For instance, Schiffman et al. optimized 11 DFT-level adsorption geometries for the organometallic dye N719 on the anatase (101) surface^18^. Among the tested geometries, the most stable configurations were those involving a double anchor. Additionally, a strong dependence of the relative stabilities on the presence or absence of surface protons, resulting from dye dissociation, was observed. In another experimental-theoretical study, Jiang et al. analyzed the anchoring of N719 on the (101), (100), and (001) surfaces of anatase doped with niobium (Nb) using DFT and subsequently evaluated the efficiency of DSSC prototypes fabricated with these materials^22^. The study yielded several noteworthy conclusions. First, Nb doping lowers the material’s work function, thereby enhancing electron injection from the dye into the semiconductor. Secondly, the incorporation of the dopant increased the semiconductor’s electron density, thereby improving conductivity. Lastly, the DSSC prototypes demonstrated a relative efficiency increase of up to 22% when Nb-doped anatase nanoparticles were used compared to undoped anatase. This improvement was attributed to the doping-induced enhancement of both the short-circuit current and the open-circuit voltage. Finally, it is worth noting that no theoretical studies have been published to date that model the adsorption of organometallic dyes on TiO_2_(B) surfaces. Kong et al. conducted a combined theoretical and experimental study investigating the interaction between an organometallic molecule with a cobalt center (Co(NH_3_)_5_Cl) and ultrathin TiO_2_(B) surfaces, aiming to enhance the photocatalytic production of hydrogen^25^. However, this study does not provide sufficient details about the parameters used in the theoretical simulations.

The primary objective of this study is to investigate the adsorption mechanism of the N719 dye on pristine TiO_2_(B) (100) surfaces using density functional theory (DFT) and to examine the effects of superficial carbon doping on surface reactivity. The N719 molecule is initially adsorbed onto a pristine ultrathin TiO_2_(B) (100) surface slab in several different interaction configurations. Subsequently, the slab is doped with carbon, and the dye is re-adsorbed onto selected systems. The modifications in adsorption performance are assessed, and the possible advantages of the application in DSSCs are discussed. This study aims to enhance the understanding of the surface chemistry and optoelectronic performance of carbon-doped TiO_2_(B) and to encourage further research towards the application of this modified material in dye-sensitized solar cells and related devices.

## Computational details

All DFT calculations were performed using the projector-augmented wave (PAW) method, as implemented in the Vienna Ab Initio Simulation Package (VASP)^26–28^. The exchange-correlation term was treated using the generalized gradient approximation (GGA) with the Perdew, Burke, and Ernzerhof (PBE) functional^29,30^. The valence electrons for the following configurations were considered: Ti (3d^2^ 4s^2^), O (2s^2^ 2p^4^), B (2s^2^ 2p^1^), C (2s^2^ 2p^2^), N (2s^2^ 2p^3^), S (3s^2^ 3p^4^), Ru (4d^7^ 5s^1^), and H (1s^1^). The Kohn-Sham wavefunctions were expanded in plane waves using a cutoff energy of 400 eV, and the Brillouin zone was sampled at the gamma point with a Monkhorst-Pack 1 × 1 × 1 k-point mesh^31^. The projection operators were evaluated in the reciprocal space, and PREC was set to Accurate as suggested by VASP. Convergence criteria for total energy and forces were set to 10^−6^ eV and 0.01 eV/Å, respectively. Gaussian smearing with a 0.03 eV sigma value was employed, as recommended by VASP. However, the GGA-PBE approach often underestimates the gap energy values due to its inadequate description of the strong on-site Coulomb interactions of localized electrons in transition metal oxides^32^. To improve the electronic structure model, the Hubbard correction proposed by Dudarev et al.^33^ was introduced to correct strong electron localization in the Ti 3 d orbitals. The effective Hubbard parameter $\:\left({\mathrm{U}}_{\mathrm{e}\mathrm{f}\mathrm{f}}\:=\:\mathrm{U}\:-\:\mathrm{J}\right)\:$was set to 4.0 eV, a value found to be optimal for accurately describing both the structural parameters and electronic properties of the TiO_2_(B) bulk phase and slabs^15,34,35^. This is consistent with other DFT + U studies suggesting $\:{\mathrm{U}}_{\mathrm{e}\mathrm{f}\mathrm{f}}$ a value between 4.0 eV and 4.2 eV is appropriate for this titania polymorph^36–39^. In addition, we estimated the $\:{\mathrm{U}}_{\mathrm{e}\mathrm{f}\mathrm{f}}$ for the Ru 4 d orbitals to be 2.1 eV using the Cococcioni approach^40^. Finally, to account for dispersion forces, Grimme DFT-D3 corrections were applied^41^.

**Fig. 1:**
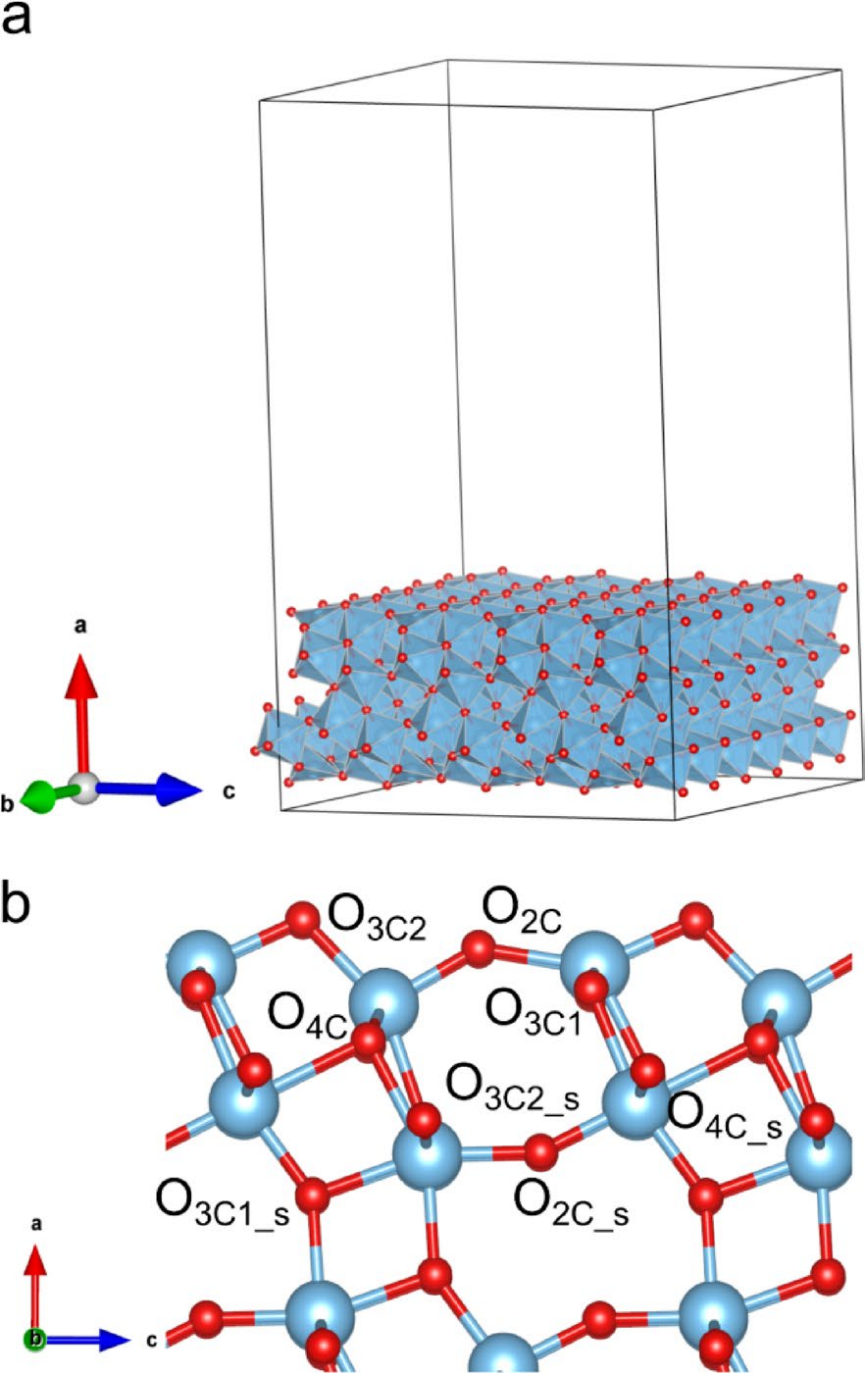
(**a**) TiO_2_(B) pristine slab employed for simulations. (**b**) Labels of the eight different O atom sites on the top two layers of the ultrathin TiO_2_(B) (100) slab according to the bulk nomenclature. Blue, Ti atoms; red, O atoms.

Periodic slabs cleaved from 6⋅6 supercells were used to model the ultrathin TiO_2_(B) (100) sheets, with a vacuum gap of approximately 30 Å along the non-periodic direction, as shown in Fig. 1a. The slabs comprised four titanium layers, resulting in 192 Ti and 384 O atoms. This configuration was chosen to balance computational efficiency with the preservation of physical properties, as no significant changes in the band gap (E_g_) and surface energy values were observed when the number of layers exceeded four^35^. As noted in a previous study^34^, oxygen sites are energetically more favorable for carbon doping than titanium sites. Consequently, after full relaxation, carbon atoms were incorporated by substituting surface and subsurface oxygen atoms at the O_3C2_ and O_4C_s_ sites, based on the bulk site nomenclature (see Fig. 1b), which constitute the preferential oxygen sites for carbon doping^15^.

N719 molecules were adsorbed on pristine and carbon-doped ultrathin TiO_2_(B) (100) surfaces (see Supplementary Fig. 1), followed by a second relaxation step of the entire system where only the position of the atoms was allowed to relax (ISIF = 2). The adsorption performance and electronic properties were then evaluated. The N719 adsorption energy was calculated using Eq. (1) and Eq. (2) for the pristine and carbon-doped ultrathin sheets, respectively:

1 $$\:{\mathrm{E}}_{\mathrm{a}\mathrm{d}\mathrm{s}}={\mathrm{E}}_{{\mathrm{T}\mathrm{i}\mathrm{O}}_{2}+\mathrm{N}719}-{\mathrm{E}}_{\mathrm{N}719}-{\mathrm{E}}_{{\mathrm{T}\mathrm{i}\mathrm{O}}_{2}}$$

2 $$\:{\mathrm{E}}_{\mathrm{a}\mathrm{d}\mathrm{s}}={\mathrm{E}}_{{\mathrm{C}-\mathrm{T}\mathrm{i}\mathrm{O}}_{2}+\mathrm{N}719}-{\mathrm{E}}_{\mathrm{N}719}-{\mathrm{E}}_{{\mathrm{C}-\mathrm{T}\mathrm{i}\mathrm{O}}_{2}}$$

where $\:{\mathrm{E}}_{{\mathrm{T}\mathrm{i}\mathrm{O}}_{2}+\mathrm{N}719}$ and $\:{\mathrm{E}}_{{\mathrm{C}-\mathrm{T}\mathrm{i}\mathrm{O}}_{2}+\mathrm{N}719}$ are the total energies of the pristine and doped slabs with a N719 molecule, respectively. $\:{\mathrm{E}}_{\mathrm{N}719}$ is the energy of the isolated molecule, while $\:{\mathrm{E}}_{{\mathrm{T}\mathrm{i}\mathrm{O}}_{2}}$ and $\:{\mathrm{E}}_{{\mathrm{C}-\mathrm{T}\mathrm{i}\mathrm{O}}_{2}}$ are the total energies of the pristine and doped slabs, respectively. According to Eqs. (1) and (2), negative $\:{\mathrm{E}}_{\mathrm{a}\mathrm{d}\mathrm{s}}$ values imply a favorable energy process. The inclusion of dipole corrections along the surface normal did not significantly alter the total energy values.

After the structural relaxation, the total and projected density of states (TDOS and PDOS) were calculated on the TiO_2_(B) (100) + N719 systems employing the correction for dispersion forces DFT-D3 and a denser k-points mesh (1 × 5 × 5). The same conditions were employed to obtain the work function approximation for pristine and carbon-doped systems. The VESTA software was used to build and visualize all the proposed models^42^. All the density of states and band structures graphics were obtained employing the Sumo Python package^43^.

While the present DFT framework reliably captures the relative adsorption strengths and electronic trends relevant to dye–semiconductor interface behavior, several inherent limitations must be acknowledged. First, absolute adsorption energies for Ru-based sensitizers can exhibit a functional dependence, particularly in the presence of multi-dentate anchoring and substantial charge redistribution. For this reason, our analysis focuses on relative trends in adsorption enhancement rather than on absolute magnitudes. Second, long-range correlation treatments and explicit electrolyte environments were not included; thus, the reported interfacial energetics should be interpreted as intrinsic surface–dye interactions in vacuum rather than full device-level energetics. Taken together, these considerations do not affect the central conclusions and establish the appropriate scope and numerical boundaries of the present computational approach.

## Results and discussion

### N719 on pristine ultrathin TiO_2_(B) (100) surface

**Fig. 2:**
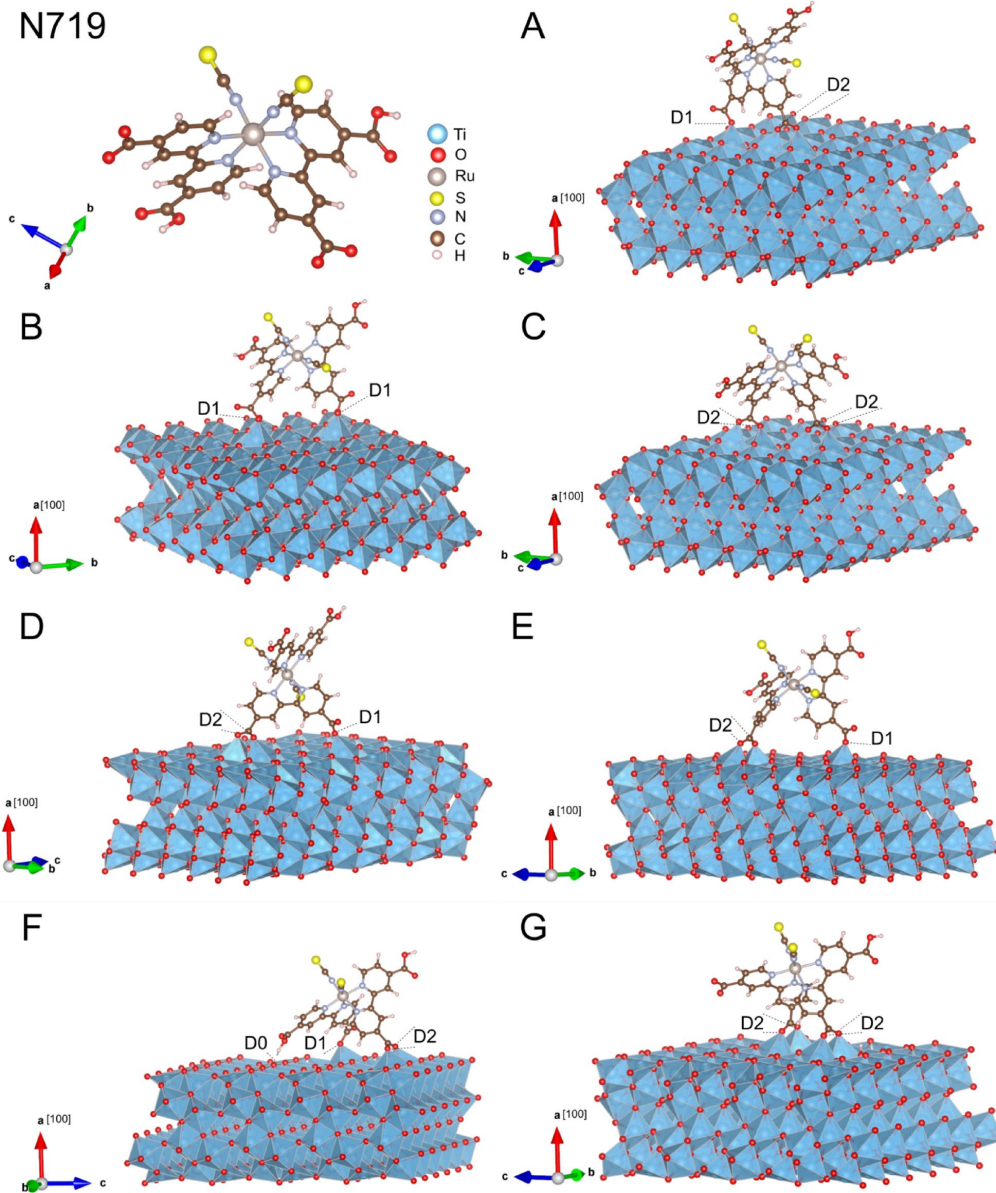
Isolated N719 molecule and 3D views of the relaxed systems (**A**-**G**) of N719 on the ultrathin TiO_2_(B) (100) surfaces with different anchoring configurations.

As reported in a previous study^35^, one of the most favorable anchoring sites for catechol dye adsorption on the TiO_2_(B) (100) surface is the pentacoordinated Ti (Ti_5C_) atoms. Consequently, it is expected that organometallic dyes such as N719 will employ their carboxylate groups to bind to these Ti_5C_ sites on the TiO_2_(B) surface. There are four different coordination types for a carboxylate group binding to a metal oxide: molecular (D0), monodentate (D1), bidentate (D2), and chelating (Ch), similar to the adsorption modes studied for the catechol molecule. The N719 molecule, however, has four carboxylate groups available for binding, two of which are protonated, as shown in Fig. 2. These carboxylate groups can adopt either trans or cis configurations relative to the thiocyanate (SCN) ligands, resulting in two possible orientations of the dye, even when the same anchoring group is used. Therefore, a wide range of adsorption geometries can, in principle, be considered.

However, based on systematic calculations of the relative energies of these configurations from previous works on anatase (101) surfaces^18,44^, the most stable geometries are anticipated to involve at least two anchoring groups, utilizing either mono- or bidentate coordination. Consequently, a limited subset of the most probable configurations for the interaction of N719 with TiO_2_(B) can be identified. The most relevant configurations, shown in Fig. 2, are described below:

- Configuration A (aligned D2 + D1): Features a bidentate and a monodentate interaction over three Ti atoms aligned in the same row.
- Configuration B (aligned 2D1): Involves two monodentate interactions on Ti atoms aligned in the same row.
- Configuration C (aligned 2D2): Consists of two bidentate interactions on aligned Ti atoms.
- Configuration D (aligned D2 + non-aligned D1): Includes one bidentate and one monodentate interaction, with the anchors aligned on different Ti atoms.
- Configuration E (aligned D2 + non-aligned D1): Comprises a bidentate interaction on aligned Ti atoms and a monodentate interaction on a non-aligned Ti atom.
- Configuration F (aligned D2 + non-aligned D1 + D0): The only system presenting three interactions: a bidentate interaction on aligned Ti atoms, a monodentate interaction on a non-aligned Ti atom, and a molecular-type interaction between an H atom of a non-dissociated carboxylate group and a surface O atom.
- Configuration G (non-aligned 2D2): Is composed of two bidentate interactions with two pairs of Ti atoms that are not aligned.

After optimizing the considered systems, the adsorption energies of the dye were calculated using Eq. 1. The results, presented in Table 1, suggest that configurations F and G are the most stable. Notably, all analyzed geometries are feasible, as most exhibit adsorption energies below −3.00 eV. This confirms the high reactivity of the (100) surface of pristine TiO_2_(B), as previously suggested by studies^45,46^. Additionally, none of the optimized structures resulted in a chelating-type binding, in concordance with a previous DFT study in which a formic acid molecule was adsorbed on anatase (101) surface, and the chelating interaction was found to be energetically unstable^47^. Importantly, the configuration that provides the highest stability for the dye on the surface (F) is the only one that involves the adsorption of three of the four carboxylate groups of the N719 molecule. The other configurations involve interactions through only two of these groups. It is worth mentioning that the adsorption energies are partially enhanced by the inclusion of the dispersion correction (DFT-D3). Bond distances and energy values without DFT-D3 are shown in Supplementary Table 1.

**Table 1:** Interatomic distances (Å) and adsorption energies (E_ads_) in the different interaction configurations between the dye N719 and pristine ultrathin TiO_2_(B) (100) surface.

Bonds	N719 + TiO_2_(B) (100) systems						
	A (D2 + D1)	B (2D1)	C (2D2)	D (D2 + D1)	E (D2 + D1)	F (D2 + D1+D0)	G (2D2)
Ti−O_1_	2.21	2.03	2.26	2.18	2.17	2.12	2.15
Ti−O_2_	2.05	ࣧ	2.06	2.10	2.08	2.13	2.17
Ti−O_3_	1.99	1.99	2.05	2.05	2.00	2.00	2.06
Ti−O_4_	ࣧ	ࣧ	2.28	ࣧ	ࣧ	ࣧ	2.19
O−H	ࣧ	ࣧ	ࣧ	ࣧ	ࣧ	1.47	ࣧ
$\:{\mathrm{E}}_{\mathrm{a}\mathrm{d}\mathrm{s}}$(eV)	−3.01	−2.82	−3.50	−3.51	−3.58	−4.46	−3.92

**Fig. 3:**
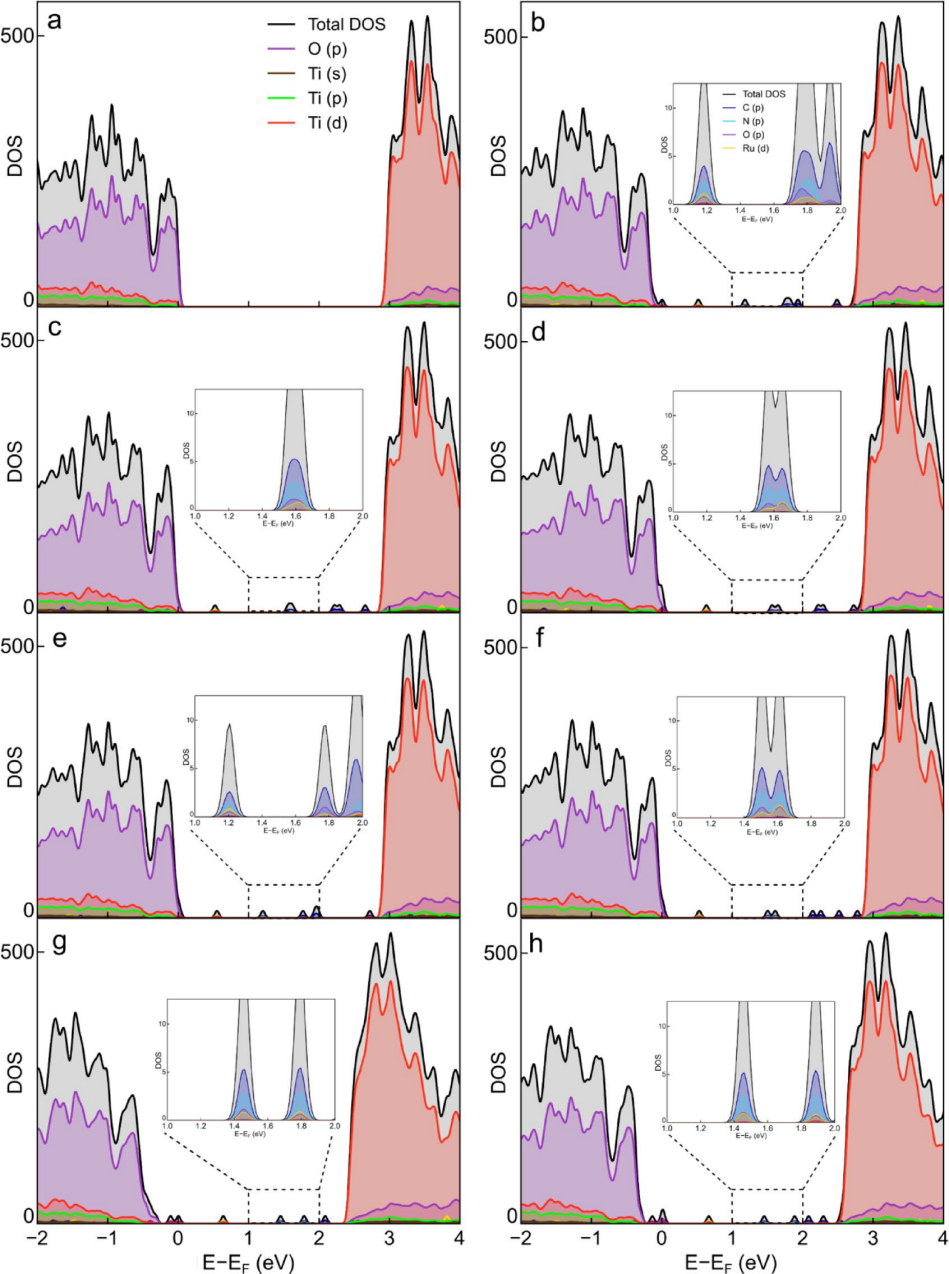
Total and partial density of states for (**a**) clean and (**b-h**) N719 adsorbed on pristine ultrathin TiO_2_(B) (100) surfaces in different interaction configurations (A-G). The inset images correspond to enlarged views of intermediate states between 1 and 2 eV.

The modifications introduced by the N719 dye on the electronic structure of the ultrathin TiO_2_(B) (100) surfaces were analyzed by computing the total and partial densities of states (DOS) depicted in Fig. 3a-h. The valence band (VB) primarily consists of O 2p states (magenta line). In contrast, the conduction band (CB) is mainly composed of Ti 3 d states (orange line). For clean pristine TiO_2_(B) (Fig. 3a), the band gap is in close agreement with the experimental value reported for TiO_2_(B) nanowires of 2.94 eV^48^. The DOS curves also reveal the presence of intermediate states within the band gap, resulting from the sensitization of the semiconductor with the N719 dye. These intermediate states near the VB, which could act as hole traps, predominantly comprise the O 2p states from the oxygen atoms in N719. In some systems, particularly in configurations F and G, these hole traps merge with the VB, leading to a reduction in the band gap. Conversely, intermediate states close to the CB, which would act as electron traps, are also detected. The composition of these electron traps varies, as shown in the insets of Fig. 3b-h. Specifically, these electron traps are made up of states associated with various atoms in the N719 dye, including Ru 4 d (yellow), O 2p (magenta), N 2p (light blue), and C 2p (blue).

In addition to the band gap energy, it is also interesting to analyze other parameters such as the Fermi energy (E_F_) and the “dye radius”, which is measured as the distance from the metallic center to the semiconductor surface. The values obtained for these parameters are presented in Table 2. Compared to the pristine surface, all N719-sensitized systems show a decrease in Eg, with systems F and G exhibiting the largest reduction of 15%. This reduction is primarily due to the introduction of intermediate states near the VB. The Fermi level of a semiconductor is typically related to the open-circuit voltage (V_OC_) in a DSSC, and consequently, to the cell’s efficiency. More negative E_F_ values are associated with higher V_OC_ values. Therefore, this criterion can be used to identify the best-performing modeled systems, as a more negative Fermi energy could result in higher efficiency. In particular, system F exhibits the lowest shift in Fermi energy (+ 0.50 eV), indicating that higher performance can be expected when the dye adopts this adsorption configuration. Additionally, a study by Klein et al., which analyzed the dynamics of photogenerated charge carriers in DSSCs using magnetic techniques, suggests that the dye radius (measured from the metal center to the surface) can serve as an indicator of the electron-hole pair lifetime^20^. Specifically, a smaller dye radius is attributed to denser surface coverage, which correlates with a lower probability of energy losses due to recombination. Therefore, based on the results obtained, it can be anticipated that a DSSC in which the N719 molecule adopts the F configuration, with a dye-TiO_2_(B) distance of 640 pm, would be more advantageous for facilitating the electron extraction process and minimizing recombination losses.

**Table 2:** Estimation of selected parameters for the N719 + TiO_2_(B) (100) systems.

Parameters	Pristine TiO_2_(B) (100)	N719 + TiO_2_(B) (100) systems						
		A	B	C	D	E	F	G
$\:{\mathrm{E}}_{\mathrm{g}}$ (eV)	2.87	2.67	2.83	2.66	2.84	2.87	2.37	2.48
$\:{\mathrm{E}}_{\mathrm{F}}$ (eV)	ࣧ	0.69	0.54	0.57	0.59	0.54	0.50	0.73
N719 radius (pm)	ࣧ	750	700	700	650	670	640	690

### N719 on ultrathin C-TiO_2_(B) (100) surface

As mentioned, carbon doping can enhance the optoelectronic properties of TiO_2_(B) and increase the reactivity of its exposed surface, thereby improving the adsorption of the sensitizer. Enhanced dye-semiconductor interactions are directly associated with greater stability and higher coverage, leading to reduced electron-hole recombination and increased light absorption. Therefore, increasing interactions with the sensitizer through carbon doping of the semiconductor is an attractive strategy for boosting the efficiency of DSSCs^49–52^. Hence, building upon the findings from the previous section regarding adsorption energies, band gap energy, Fermi energy, and N719 distance across various interaction configurations of the dye with ultrathin TiO_2_(B) films (100), this section investigates the impact of incorporating carbon impurities at the most stable sites (O_3C2_ and O_4C_s_). The analysis focuses specifically on the preferred configurations F and G of the dye.

**Fig. 4:**
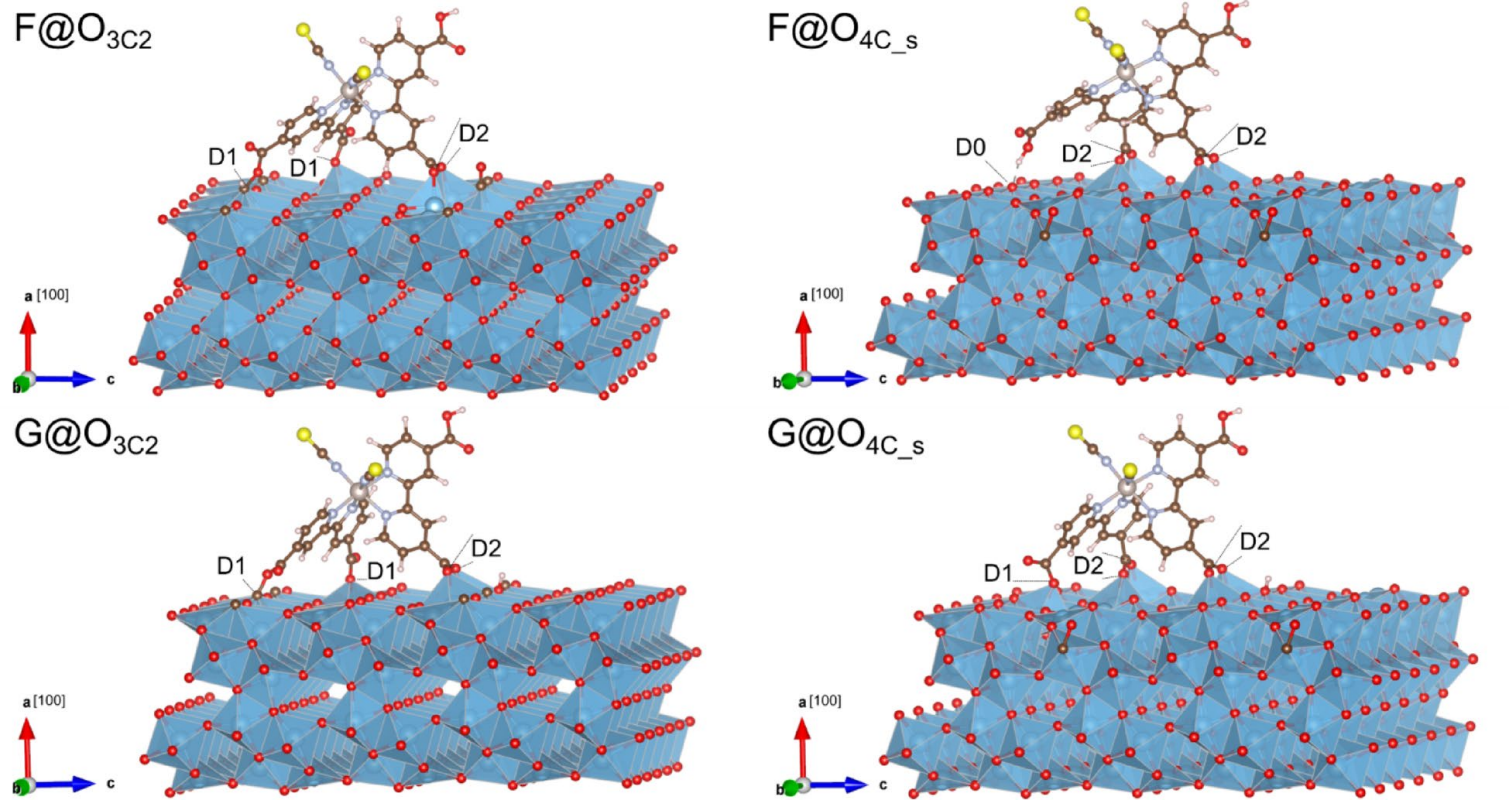
3D views of the relaxed systems (F and G) of N719 on the ultrathin C-TiO_2_(B) (100) surfaces (C@O_3C2_ and C@O_4C_s_ sites) with different anchoring configurations.

Similar to the previous analysis, Table 3 presents interatomic distances and adsorption energies computed for selected interactions between the N719 dye and the C-doped TiO_2_(B) (100) surface using Eq. (2). These configurations are illustrated in Fig. 4. Notably, all four systems examined exhibit surface anchoring via three carboxylate groups. This behavior was previously observed for the F configuration (D2 + D1+D0 anchors), representing the most stable adsorption arrangement of N719 on the pristine surface. However, in the doped systems, the dye maintains the original F interaction only for C@O_3C2_, whereas it adopts a new D2 + D2+D0 coordination for C@O_4C_s_. It can also be noted that different anchor combinations derive from the initial G configuration (two D2 interactions), featuring D2 + D1+D1 anchors for G + C@O_3C2_ and D2 + D2+D1 for G + C@O_4C_s_. Specifically, for G + C@O_3C2_, a D2 anchor is replaced by a D1 anchor, allowing the addition of another D1 anchor via a different carboxylate group. As shown in Table 3, the described coordination arrangements exhibit significant adsorption energies when carbon impurities are present on the surface, making F + C@O_3C2_ the preferred system. As already stated, the inclusion of DFT-D3 improves the stability of N719, which can be compared with considering the adsorption energies when no dispersion correction is included (refer to Supplementary Table 2). It is worth mentioning that, when normalizing the adsorption energy per carboxylate anchor, the absolute values are well aligned with those reported in the literature^17–25^.

Additionally, it is noteworthy that all systems exhibit higher E_ads_ values compared to those obtained on the pristine surface (see Table 1). The results suggest that incorporating this dopant enhances the reactivity of TiO_2_(B) surfaces, potentially improving the performance of DSSCs based on carbon-doped titania B, particularly in terms of long-term stability. Furthermore, the dye radius decreases for the four doped systems compared to the values obtained in the pristine surface. This behavior suggests more efficient extraction of photogenerated electrons, which could further improve the overall performance of DSSCs.

**Table 3:** Interatomic distances (Å), adsorption energy between the N719 dye and the ultrathin C-TiO_2_(B) (100) surface, and N719 radius.

Bonds	N719 + C-TiO_2_(B) systems			
	F + C@O_3C2_	F + C@O_4C_s_	G + C@O_3C2_	G + C@O_4C_s_
Ti−O_1_	2.08	2.10	2.11	2.12
Ti−O_2_	2.14	2.14	2.16	2.15
Ti−O_3_	1.97	2.18	2.03	1.99
Ti−O_4_	ࣧ	2.50	2.16	2.22
Ti−O_5_	ࣧ	ࣧ	ࣧ	2.08
O−H	ࣧ	ࣧ	1.67	ࣧ
C−O	1.42	1.34	ࣧ	ࣧ
$\:{\mathrm{E}}_{\mathrm{a}\mathrm{d}\mathrm{s}}$(eV)	−11.33	−7.30	−9.29	−6.12
$\:\mathrm{E}$(eV/carboxylate)	−3.78	−2.43	−3.10	−2.04
N719 radius (pm)	605	640	595	615

### Implications of C-TiO_2_(B) as electrode for DSSCs

The overall performance of dye-sensitized solar cells depends on the binding of dye molecules to semiconductor surfaces. As shown in previous sections, N719 dye exhibits favorable adsorption to pristine ultrathin TiO_2_(B) (100) surfaces at an energy level of approximately 3 eV. Still, this interaction becomes much stronger (up to −11 eV) after surface carbon doping. The observed improvement in coupling between the dye’s electronic states and the surface conduction band suggests that the electron injection efficiency and rate will increase.

**Fig. 5:**
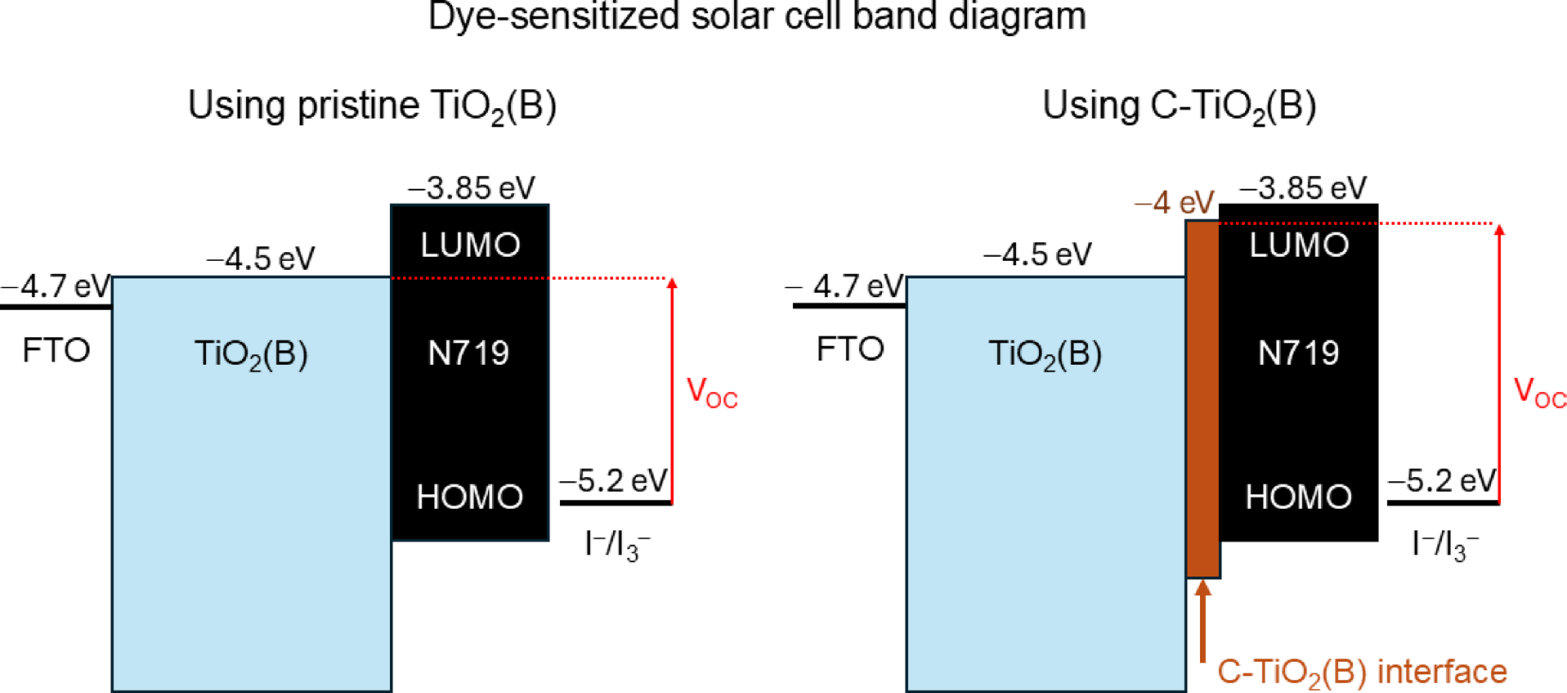
Representation of the band diagram of a DSSC employing pristine TiO_2_(B) (left) and C-TiO_2_(B) (right) as semiconductors.

Carbon doping results in a clear reduction of the work function (WF) relative to the pristine surface, as depicted in the Supplementary Fig. 2, indicating an upward shift of the Fermi level with respect to the vacuum level^53^. This behavior is consistent with the enhanced donor character introduced by carbon substitution. Yet, without changing the fundamental absorption behavior present in TiO_2_(B) (direct absorption) as seen in the Supplementary Fig. 3. From a DSSC perspective, a lower work function favors electron injection from the excited N719 dye into the TiO_2_ conduction band and can enhance charge separation at the dye–semiconductor interface. Additionally, C-TiO_2_(B) enhances dye anchoring and provides additional benefits that can affect DSSC photovoltaic parameters, such as the open-circuit voltage and the photocurrent density (J_SC_). The V_OC_ of DSSCs depends on the difference between the redox potential (e.g., I⁻/I_3_⁻) and the quasi-Fermi level of electrons (E_Fn_) in TiO_2_ under illumination, as displayed in Fig. 5. This is determined by the equilibrium between the rates of electron injection and electron recombination. The conduction band edge of TiO_2_ serves as the maximum potential for V_OC_, as it is improbable that the quasi-Fermi level surpasses the CB edge.

Since carbon doping is thermodynamically favored at surface sites, it is reasonable to assume that the bulk electronic structure of TiO_2_(B) remains unchanged, preserving the original conduction band minimum (CBM) position. In this way, the V_OC_ ceiling could remain identical to pristine TiO_2_(B) as the CBM of the TiO_2_(B) bulk material stays unaffected. The surface-localized electronic states created by carbon doping form “stepping stones” that connect the dye’s lowest unoccupied molecular orbital (LUMO) to the TiO_2_ conduction band, providing favorable energy pathways. This structural configuration enables rapid and efficient dye-to-surface conduction-band charge transfer, allowing electrons to enter the semiconductor bulk^54^. The separation of injection and transport mechanisms across space and energy domains speeds up charge injection into the oxide and reduces electron recombination at the dye–electrolyte interface^55^.

These factors are vital when determining the quasi-Fermi level at open-circuit conditions. Considering the diode equation, it shows that both increases in electron injection rate and reductions in recombination rate result in a V_OC_ increase of approximately 60 mV per order of magnitude^56^. While the absolute V_OC_ is ultimately limited by the conduction band minimum of bulk TiO_2_(B), even modest shifts in recombination dynamics can produce measurable improvements. The enhanced dye-surface coupling indicates improved electron injection efficiency, resulting in a higher photocurrent by maximizing photon absorption.

The obtained band gap reduction from 2.90 eV in pristine to 2.37 eV in C-TiO_2_(B) suggests that an improvement is feasible due to the alignment of the dye’s LUMO with TiO_2_ CB and the visible light absorption of the oxide surface. The improved electronic overlap and charge transfer kinetics of C-TiO_2_(B) suggest that this material could be suitable for future DSSC applications, even though light-harvesting improvements may not be as significant as dye absorption.

## Conclusions

In this study, the adsorption of the N719 dye on pure and C-doped ultrathin TiO_2_(B) (100) films was evaluated using DFT + U methods to determine whether the introduction of the dopant enhances the applicability of this semiconductor in DSSC systems. Calculations enabled the determination of seven stable anchoring configurations of N719 on the pristine surface, with the most stable configuration occurring when the interaction occurs through three of the four carboxylate groups. On the other hand, carbon doping at surface sites was also found to significantly increase the interaction between the sensitizer and the semiconductor (up to 300%), particularly when the dye anchors to the surface via three carboxylate groups. This may lead to improved long-term stability of DSSCs. Additionally, the density of states curves allowed us to verify that incorporating carbon impurities induces hybrid interfacial states, while also reducing the bandgap energy and the distance of the dye from the surface, thereby optimizing the material’s properties for light absorption and carrier transport. These insights could guide future experimental synthesis of C-doped TiO_2_(B) electrodes with enhanced dye anchoring.

## Supplementary Information

Below is the link to the electronic supplementary material.

Supplementary Material 1

## Data Availability

The datasets used and/or analyzed during the current study are available from the corresponding author upon reasonable request.
