# Supplementary material for: Computational study of carbon-doped TiO2(B) nanomaterials for improved dye-sensitized solar cells
Source: Sci Rep. 2026 Feb 10;16:8180. doi: 10.1038/s41598-026-38897-7 (PMC12963604; doi:10.1038/s41598-026-38897-7)
Supplement: Supplementary file 1 — Supplementary Material 1 [file 41598_2026_38897_MOESM1_ESM.docx]

**Supplementary Information**

Supplementary Tables

**Supplementary Table 1:** Interatomic distances (Å) and adsorption energies (E_ads_) in the different interaction configurations between the dye N719 and pristine ultrathin TiO_2_(B) (100) surface without vdW dispersion correction (No DFT-D3).

|  | **Bonds** | **TiO_2_(B) (100) +N719 no vdW systems (no DFT-D3)** | | | | | | |  |
| --- | --- | --- | --- | --- | --- | --- | --- | --- | --- |
|  |  | **A (D2+D1)** | **B (2D1)** | **C (2D2)** | **D (D2+D1)** | **E (D2+D1)** | **F (D2+D1+D0)** | **G (2D2)** |  |
|  | Ti−O_1_ | 2.21 | 2.03 | 2.26 | 2.18 | 2.18 | 2.12 | 2.17 |  |
|  | Ti−O_2_ | 2.05 | ⎯ | 2.06 | 2.10 | 2.09 | 2.14 | 2.18 |  |
|  | Ti−O_3_ | 2.00 | 1.99 | 2.05 | 2.05 | 2.00 | 2.02 | 2.06 |  |
|  | Ti−O_4_ | ⎯ | ⎯ | 2.29 | ⎯ | ⎯ | ⎯ | 2.22 |  |
|  | O−H | ⎯ | ⎯ | ⎯ | ⎯ | ⎯ | 1.48 | ⎯ |  |
|  | $E_{\mathrm{ads}}$(eV) | −2.58 | −2.38 | −3.00 | −2.83 | −3.00 | −3.57 | −3.30 |  |
|  |  |  |  |  |  |  |  |  |  |

**Supplementary Table 2:** Interatomic distances (Å), adsorption energy between the N719 dye and the ultrathin C-TiO_2_(B) (100) surface without vdW dispersion correction (No DFT-D3).

|  | **Bonds** | C**-TiO_2_(B) + N719 no vdW systems (no DFT-D3)** | | | |  | |
| --- | --- | --- | --- | --- | --- | --- | --- |
|  |  | **F**+**C @O_3C2_** | **F**+**C @O_4C_sub_** | **G**+**C @O_3C2_** | **G**+**C @O_4C_sub_** |  |  |
|  | Ti−O_1_ | 2.02 | 2.11 | 2.13 | 2.12 |  |  |
|  | Ti−O_2_ | 2.17 | 2.16 | 2.11 | 2.14 |  |  |
|  | Ti−O_3_ | 2.00 | 2.04 | 2.20 | 2.07 |  |  |
|  | Ti−O_4_ | ⎯ | 2.15 | ⎯ | 2.12 |  |  |
|  | O−H | ⎯ | 1.59 | ⎯ | ⎯ |  |  |
|  | C−O | ⎯ | ⎯ | 1.33 | ⎯ |  |  |
|  | C−H | 2.17 | ⎯ | ⎯ | ⎯ |  |  |
|  | $E_{\mathrm{ads}}$(eV) | −5.59 | −4.53 | −6.20 | −4.55 |  |  |

Supplementary Figures


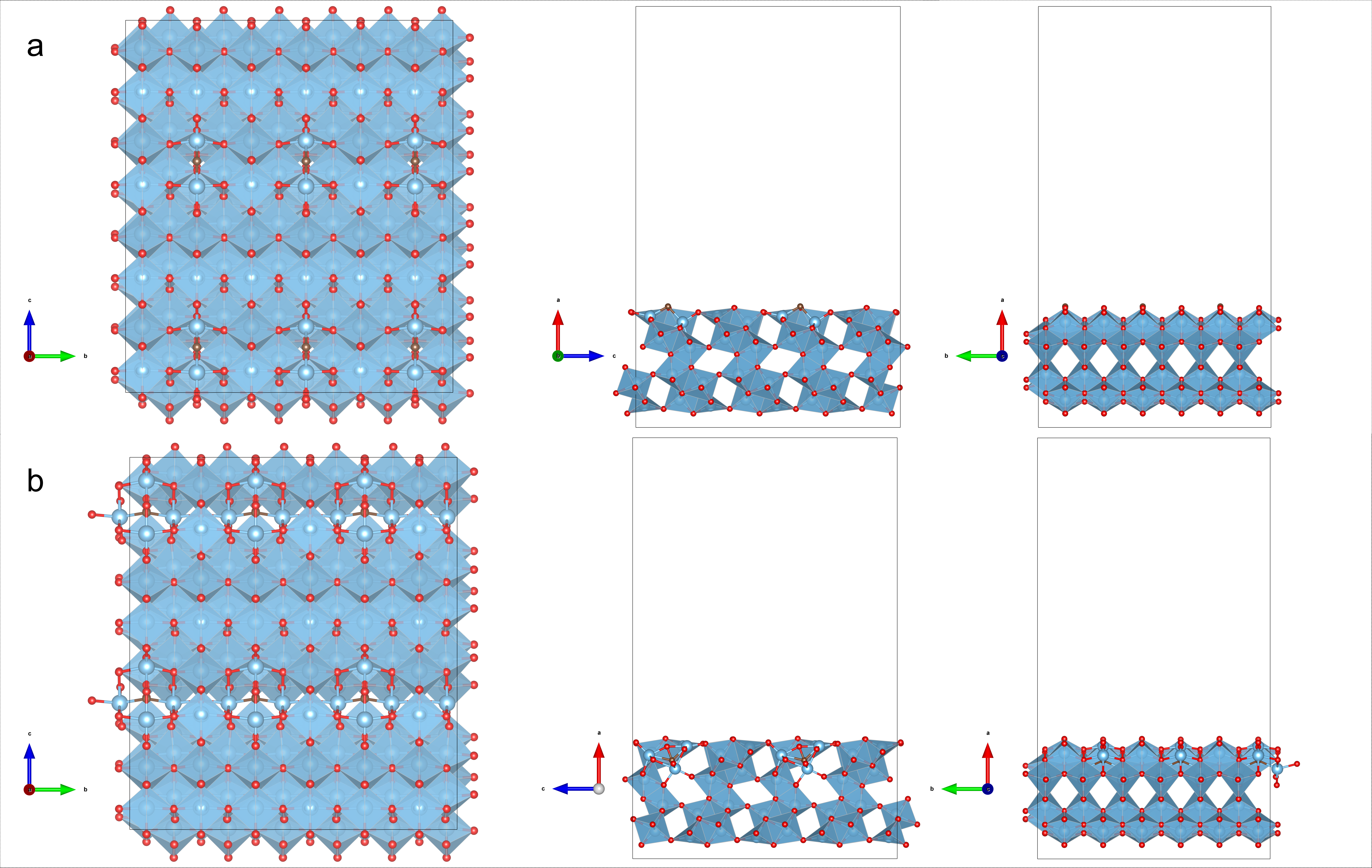


**Supplementary Figure 1:** Top and side views of C-TiO2(B) (100) for a) O_3C2_ and b) O_4C_s_ relaxed systems.


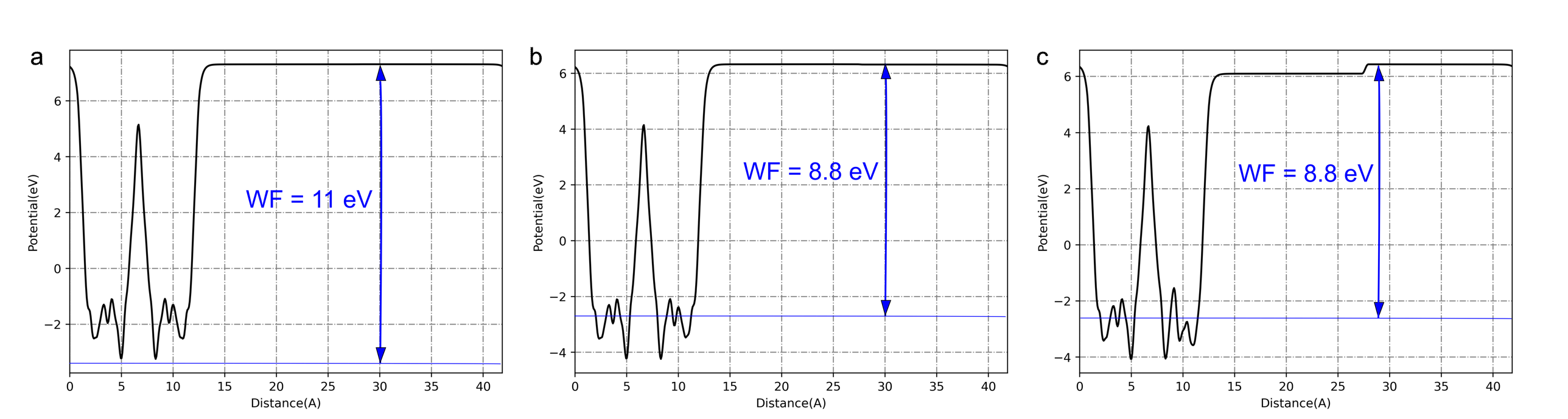


**Supplementary Figure 2:** Potential energy with the estimated work function for a, Pristine TiO_2_(B) (100), b, O_3C2_ and c, O_4C_s_ systems.


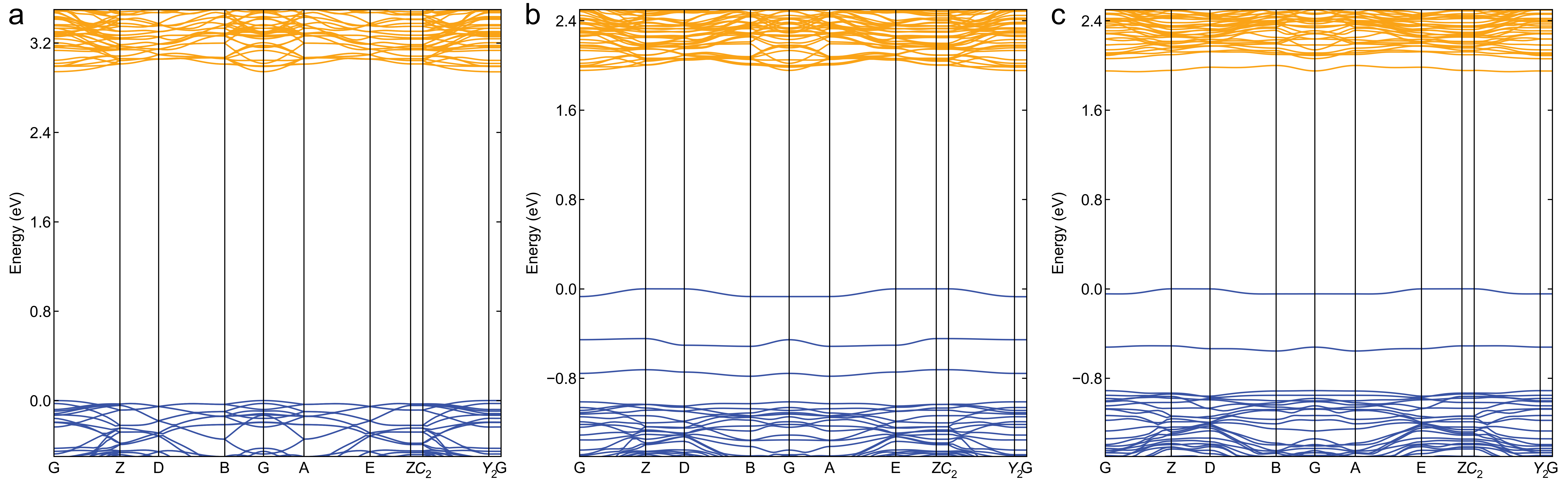


**Supplementary Figure 3:** Calculated band-structure for a, Pristine TiO_2_(B) (100), b, O_3C2_ and c, O_4C_s_ systems.
